# Assessment of Arabian Gulf Seaweeds from Kuwait as Sources of Nutritionally Important Polyunsaturated Fatty Acids (PUFAs)

**DOI:** 10.3390/foods10102442

**Published:** 2021-10-14

**Authors:** Hanan Al-Adilah, Tahani Khalaf Al-Sharrah, Dhia Al-Bader, Rainer Ebel, Frithjof Christian Küpper, Puja Kumari

**Affiliations:** 1 School of Biological Sciences, University of Aberdeen, Cruickshank Building, St. Machar Drive, Aberdeen AB24 3UU, UK; h.aladilah.17@abdn.ac.uk (H.A.-A.); fkuepper@abdn.ac.uk (F.C.K.); 2 Marine Biodiscovery Centre, Department of Chemistry, University of Aberdeen, Aberdeen AB24 3UE, UK; r.ebel@abdn.ac.uk; 3 Environmental Pollution & Climate Program, Environment and Life Sciences Research Centre, Kuwait Institute for Scientific Research, P.O. Box 24885, Safat 13109, Kuwait; t.sharrah@kisr.edu.kw; 4 Plant Biology Program, Department of Biological Sciences, Faculty of Science, Kuwait University, P.O. Box 5969, Safat 13060, Kuwait; dhiaaibader@yahoo.com

**Keywords:** Arabian Gulf, fatty acids, gas chromatography, *n*6/*n*3 ratio, PUFA, seaweed

## Abstract

The fatty acid (FA) compositions of ten seaweeds representative of Chlorophyta, Rhodophyta, and Ochrophyta from Kuwait in the Arabian Gulf region were determined and are discussed in the context of their potential nutritional perspectives for seaweed valorization. All the seaweeds had higher saturated fatty acid (SFA) and lower monounsaturated (MUFA) and polyunsaturated fatty acid (PUFA) contents than those typical of tropical environments. Palmitic, myristic, stearic, oleic, linoleic, α-linolenic, and stearidonic acids were the major FAs detected. Arachidonic, eicosapentaenoic, and docosahexaenoic acids were detected in minor amounts. Conserved fatty acid patterns revealed phylogenetic relationships among phyla, classes, and orders matching the molecular phylogenies at higher taxonomic ranks. Hierarchical clustering analyses clearly segregated different seaweeds (except *Codium papillatum* and *Iyengaria stellata*) into distinct groups based on their FA signatures. All but one species (*Chondria* sp.) had health-beneficial *n*6/*n*3 PUFAs (0.33:1–2.94:1) and atherogenic (0.80–2.52) and thrombogenic indices (0.61–5.17). However, low PUFA/SFA contents in most of the species (except *Ulva* spp.) may limit their utilization in the formulation of PUFA-rich functional foods. *Ulva* spp. had substantially high PUFAs with PUFA/SFA > 0.4, *n*6/*n*3 (0.33–0.66) and atherogenic (0.80–1.15) and thrombogenic indices (0.49–0.72), providing substantial potential for their utilization in food and feed applications.

## 1. Introduction

Seaweeds are photosynthetic, multicellular marine macroalgae that have been utilized for food, animal feed, phycocolloids, and bioactive compounds of pharmacological importance for centuries. In fact, they are considered one of the most important food sources for the coastal communities especially in Asian countries such as Japan, China, and Korea [1]. Today the global seaweed industry is worth USD 6 billion per annum, 85% of which comprise food products for human consumption [1]. These seaweeds are rich sources of essential nutrients and health-promoting compounds including proteins, carbohydrates, polyunsaturated fatty acids (PUFAs), antioxidants, minerals, dietary fibers, and vitamins [2,3,4,5]. As a matter of fact, it is often pointed out that the Japanese, who have eaten seaweeds regularly in their daily cuisines for centuries, have one of the highest life expectancies in the world [6]. Seaweed-digesting enzymes such as porphyranases and agarases were discovered in the Japanese gut bacteria a decade ago, but were absent from American populations [7]. Moreover, increasing awareness of beneficial impacts of seaweed-based food products for health and their popularization in Western and European markets have recently opened a debate on categorizing seaweeds as a ‘healthy superfood’ [8,9,10].

Nevertheless, seaweeds feature low lipid contents (<5% d.w.) [2,4,11,12,13]. Seaweed lipids have received considerable interest due to their high contents of nutritionally essential *n*3 and *n*6 PUFAs [3,11,14,15] that cannot be synthesized by humans and are thus obtained only through dietary sources. Moreover, seaweeds also synthesize long-chain PUFAs (LC-PUFAs) such as arachidonic acid (ARA), eicosapentaenoic acid (EPA), and docosahexaenoic acid (DHA), which are not present in land plants [2,4,11,14,15,16,17]. These essential LC-PUFAs are physiologically important and are involved in the regulation of membrane structure and function, transcription regulation, cell signaling, and generation of bioactive lipid mediators such as prostaglandins [18]. Among LC-PUFAs, *n*3 PUFAs are of immense clinical and nutritional importance since a balance of *n*6 and *n*3 PUFAs is critical for the prevention of chronic diseases including cardiovascular diseases, colon and breast cancers, neurodegenerative and inflammatory diseases, and is also crucial for infant brain development [18,19,20,21,22,23]. Numerous seaweeds have been recognized to have health-promoting effects reflected in their nutritional indices such as *n*6/*n*3 PUFA ratio (0.1:1–3:1), as well as an atherogenic index (AI) and thromogenic index (TI) of both less than one [2,4,11,14,24,25], underlining their potential utilization in nutritional and functional food formulations. AI signifies the relationship between the sum of pro-atherogenic saturated fatty acids (SFAs) such as 12:0, 14:0, and 16:0 and anti-atherogenic unsaturated fatty acids (UFAs) [23,26]. The pro-atherogenic FAs favor the adhesion of lipids to cells of the immunological and circulatory system while anti-atherogenic FAs inhibit the aggregation of plaques and reduce the levels of esterified fatty acid, cholesterol, and phospholipids, thereby preventing the appearance of micro- and macro- coronary diseases [23,27]. TI signifies the relationship between the pro-thrombogenic FAs (12:0, 14:0, and 16:0) and anti-thrombogenic monounsaturated fatty acids (MUFAs), *n*3 and *n*6 PUFAs [26]. The dietary intake of food with low AI and TI reduces the threat of plaque formation and of atrial fibrillation respectively, thereby improving cardiovascular health [23].

Furthermore, seaweed FA profiles represent distinct conserved chemotaxonomic traits that have been extensively used to classify seaweeds to different taxonomic levels of genus, family, order, and phylum [4,11,14,15,17]. The taxon-specific understanding of FA profiles of seaweeds is necessary for selecting appropriate seaweed taxa for their valorization as human food, animal feed, or development of other nutraceutical, pharmaceutical, and cosmeceutical products [4,14]. Therefore, it is not surprising that numerous seaweeds have been studied from different parts of the world for their fatty acids and this dataset is continuously growing [15,25,28,29,30]; still, the absolute number of seaweeds studied is low considering the overall seaweed diversity, with approximately 10,000 species been reported worldwide [31]. 

The Arabian Gulf is a shallow basin located in one of the most arid regions of the world. The coastal environment of Kuwait (approximately 500 km coastline) can be divided into the Northern Region, Kuwait Bay, and the Southern Region. Marine organisms of this region experience the greatest seasonal temperate range in the world as well as the highest annual sea temperature [32] along with high levels of salinity (40–41 g/kg). The unique extreme environmental conditions may well be associated with unusual fatty acid profiles, but surprisingly, only a few seaweeds from the Arabian Gulf coast of Qatar [33], Saudi Arabia [34], and Iran [35,36,37,38] have been studied in this context to date and, in particular, information on FA profiles of seaweeds from hypersaline and warm coastal waters of Kuwait is mostly lacking so far. A notable exception dating back almost two decades is the pioneering study by Al-Hasan et al. [39]. In the present study, we analyzed FA composition by gas chromatography with flame ionization detection (GC-FID) of ten seaweeds exhibiting widespread distribution and high abundance in Kuwait coastal waters, belonging to the genus *Ulva*, *Codium*, *Chondria*, *Iyengaria*, *Feldmannia*, *Padina*, and *Sargassum*. Our aim was to identify seaweed species containing high levels of nutritionally essential PUFAs that can potentially be valorized for human consumption or other functional food applications. In this pursuit, we also studied FA-based conserved taxonomic differences among different species using hierarchical clustering.

## 2. Materials and Methods

### 2.1. Seaweed Sample Collection

A total of 10 different seaweed species, *Ulva* sp.*, Ulva chaugulii* M. G. Kavale and M. A. Kazi, *Ulva ohnoi* M. Hiraoka and S. Shimada, *Ulva* tepida Y. Masakiyo and S. Shimada, *Codium papillatum* C. K. Tseng and W. J. Gilbert (Chlorophyta), *Chondria* sp. C. Agardh (Rhodophyta), *Iyengaria stellata* (Børgesen) Børgesen, *Feldmannia indica* (Sonder) Womersley and A. Bailey, *Padina boergesenii* Allender and Kraft, and *Sargassum aquifolium* (Turner) C. Agardh (Ochrophyta) were collected during May to June 2018 and in February 2021 from different sampling sites of Kuwait’s coastal waters in the Arabian Gulf (Table 1). Seaweed samples were rinsed thoroughly with seawater on-site and placed in plastic bags. Date of collection and location were noted. Samples were transferred to the laboratory in cool packs and washed thrice with sea water. Fresh samples were frozen at −20 °C for 24 h followed by freeze-drying in a freeze dryer (Labconco, Kansas City, MO, USA) at −45 °C for 48 h and stored at −20 °C until analysis. 

### 2.2. Fatty Acid Extraction and Methyl Ester Preparation

Fatty acids were extracted and converted into the respective methyl esters from freeze-dried samples by the base-catalyzed direct transmethylation method modified after Christie and Han [40]. Briefly, 0.3 g of freeze-dried seaweed samples (in triplicates) were homogenized in a mortar and pestle and transferred to Oakridge™ centrifuge tubes (15 mL), to which 3 mL of KOH-MeOH solution (0.2 M) was added. The mixture was heated at 75 °C for 1 h. After cooling to room temperature, 3 mL of n-hexane was added and mixed thoroughly using a vortex. The organic layers containing fatty acid methyl esters (FAMEs) were collected in GC vials and stored at −20 °C until analysis.

### 2.3. Gas Chromatographic (GC) Analysis

For analysis of FAMEs, 1 µL of esterified sample was injected into a gas chromatograph (Shimadzu GC 2010, Tokyo, Japan) coupled with a flame ionization detector (FID). A cyano-polysiloxane (CP-Sil 88 for FAME, part number 839171) capillary column (100 m × 0.25 mm, 0.20 µm (J&W, Varian, Chrompack, São Paulo, Brazil) was used for the FAMEs separation under the following instrumental conditions: injector and FID detector temperatures were 250 and 270 °C, respectively, with an injector split ratio of 1:50, and carrier gas helium with a constant flow rate of 1.0 mL/min. The initial oven temperature was 80 °C, at which it was held for 5 min, followed by an increase to 220 °C at a rate of 4 °C/min; then, it was held for 5 min, and finally the temperature was increased to 240 °C at a rate of 1 °C/min and was held for an additional 10 min. FAME peaks were identified by comparison of their retention times with those of external standard (FAME Mix C4-C24; Sigma-Aldrich, Laramie*,* WY*,* USA) and quantified by area normalization using postrun analysis, GC LabStationsTM software v. 5.96 (Shimadzu, Tokyo, Japan). The content of individual fatty acid was finally reported as relative percentage of the total fatty acid methyl esters (TFAs).

### 2.4. Nutritional Indices

The unsaturation index (U.I.) was calculated by multiplying the percentage of each fatty acid by the number of double bonds followed by summing up their contributions [41]. Atherogenic and thrombogenic indices (AI and TI) were calculated according to Ulbright and Southgate [26], where:

AI = (12:0 + 4 × 14:0 + 16:0)/(*n* − 3 PUFAs + *n* − 6 PUFAs + MUFAs), and

TI = (14:0 + 16:0 + 18:0)/(0.5*n* − 6 PUFAs + 3*n* − 3PUFAs + *n*3/*n* − 6 PUFAs)

### 2.5. Statistical Analysis

All analytical determinations were performed in triplicate (*n* = 3) and the mean values were recorded. The fatty acid contents of different seaweed species were compared by analysis of variance (ANOVA) followed by Tukey’s HSD post-hoc test with differences considered significant at *p* < 0.01 using SPSS v 22. All multivariate analyses were performed after log-transformation and pareto scaling (mean-centered and divided by the square root of standard deviation of each value) of FA and nutritional data matrices (Supplementary datasheet S1–S2) using the web-based software MetaboAnalyst v 5.0 (https://www.metaboanalyst.ca accessed on 26 September 2021). This data pre-processing was carried out to give equal weight to all variables, regardless of their absolute value as the detected fatty acid levels were of different orders of magnitude. The principal component analysis (PCA) was performed on data matrices without rotation and the principal components were extracted based on scree plot. The dendrogram was obtained by hierarchical clustering based on Ward linkage with Euclidean distance [42]. Additionally, the normalized data matrices obtained in MetaboAnalyst were exported to SPSS v 22 for Kaiser-Meyer-Olkin (KMO) test for measuring sampling adequacy for PCA analysis and Bartlet’s test of sphericity to assess the equality of variance in the data matrices.

## 3. Results and Discussion

### 3.1. Fatty Acid Composition

A total of 31 fatty acids were detected in seaweeds using GC-FID listed in Table 2. SFAs constituted of decanoic acid (10:0), dodecanoic acid (12:0), tridecanoic acid (13:0), tetradecanoic acid (14:0; myristic acid), pentadecanoic acid (15:0), hexadecanoic acid (16:0; palmitic acid), heptadecanoic acid (17:0), octadecanoic acid (18:0; stearic acid), icosanoic acid (20:0; arachidic acid), docosanoic acid (22:0; behenic acid), and tetracosanoic acid (24:0; lignoceric acid). MUFAs constituted (9*Z*)-tetradec-9-enoic acid (9c-14:1; myristoleic acid), (10*Z*)-pentadec-10-enoic acid (10c-15:1), (9*Z*)-hexadec-9-enoic acid (9c-16:1; palmitoleic acid), (9*Z*)-octadec-9-enoic acid (9c-18:1; oleic acid), (9*E*)-octadec-9-enoic acid (9t-18:1; elaidic acid), (11*Z*)-icos-11-enoic acid (11c-20:1; gondoic acid), (13*Z*)-docos-13-enoic acid (13c-22:1; erucic acid), and (15*Z*)-tetracos-15-enoic acid (15c-24:1; nervonic acid). PUFAs constituted (9*Z*,12*Z*)-octadeca-9,12-dienoic acid (9c12c-18:2; linoleic acid, LA), (9*E*,12*E*)-octadeca-9,12-dienoic acid (9t12t-18:2; linolelaidic acid), (6*Z*,9*Z*,12*Z*)-octadeca-6,9,12-trienoic acid (6c9c12c-18:3; γ-linolenic acid, GLA), (9*Z*,12*Z*,15*Z*)-octadeca-9,12,15-trienoic acid (9c12c15c-18:3; ɑ-linolenic acid, ALA), (6*Z*,9*Z*,12*Z*,15*Z*)-octadeca-6,9,12,15-tetraenoic acid (6c9c12c15c-18:4; stearidonic acid, STA), (8*Z*,11*Z*,14*Z*)-icosa-8,11,14-trienoic acid (8c11c14c-20:3; dihomo-γ-linolenic acid, DGLA), (11*Z*,14*Z*,17*Z*)-icosa-11,14,17-trienoic acid (11c14c17c-20:3; dihomolinolenic acid), (5*Z*,8*Z*,11*Z*,14*Z*)-icosa-5,8,11,14-tetraenoic acid (5c8c11c14c-20:4; arachidonic acid, ARA), (5*Z*,8*Z*,11*Z*,14*Z*,17*Z*)-icosa-5,8,11,14,17-pentaenoic acid (5c8c11c14c17c-20:5; eicosapentaenoic acid, EPA), (13*Z*,16*Z*)-docosa-13,16-dienoic acid (13c16c-22:2), and (4*Z*,7*Z*,10*Z*,13*Z*,16*Z*,19*Z*)-docosa-4,7,10,13,16,19-hexaenoic acid (4c7c10c13c16c19c-22:6; docosahexaenoic acid, DHA). The fatty acid contents of seaweeds stated here refer to relative contribution to total fatty acids (% TFA) throughout the manuscript. 

#### 3.1.1. Chlorophyta

The major FAs detected in Chlorophyta species were 16:0, 18:0, 9c-16:1, 9c-18:1, 9c12c-18:2, 9c12c15c-18:3, and 6c9c12c15c-18:4, which together accounted for 81.5% to 86.2% of TFA. The contents of SFAs were high (approximately 44.5% to 51.6% in *Ulva* spp. to 72.0% in *C*. *papillatum*). The contents of MUFAs were low (11.5% in *U*. *ohnoi* to 20.7% in *U*. *tepida*), while PUFAs ranged from 29.2% in *U*. *chaugulii* to 35.4% in *U*. *tepida*, except for *C*. *papillatum*, which had exceptionally low PUFAs (7.9%) (Table 2).

They had a characteristic FA profile of higher C18 PUFAs than C20 PUFAs, 11.2 to 21.8-fold higher in *Ulva* spp. and 3.0-fold higher in *C*. *papillatum* in congruence with previous studies [4,11,14,15,43,44,45,46,47,48]. The contents of long-chain PUFAs 5c8c11c14c-20:4 and 5c8c11c14c17c-20:5 were considerably lower as compared with red and brown seaweeds (1.2–2.2%) (Table 2). The FA profiles of green seaweeds from Arabian Gulf in the present study were very similar to those reported from tropical or sub-tropical regions of warm climate, exhibiting high SFA and low MUFA and PUFAs [11,35,43,44,45,46].

Further, the members of the same genus exhibited similar FA patterns but differed significantly in their individual FA contents (*p* < 0.01) as reported in previous studies [4,11,14]. *Ulva* spp. displayed characteristic FA profiles of high 16:0, 9c-18:1, C18 PUFAs with higher 9c12c15c-18:3 content than 9c12c-18:2 (1.6- to 2.6- times, except in *U*. *ohnoi*), and low C20 PUFAs, as reported previously for the same or related *Ulva* spp. [4,14,15,35,39,46,49]. However, exceptionally higher contents of MUFAs (29.1–32.37%), especially of 9c-18:1 (22.3–26.8%), which had approximately 1.4 to 4.6-fold higher values than the present study, have been reported previously for *Ulva* species from Iran [35,43].

6c9c12c15c-18:4 is another characteristic FA reported for *Ulva* spp. [4,14,25,46,49]. *Ulva* spp. displayed the highest content of 6c9c12c15c-18:4 among all the seaweeds in our study, approximately 5.6% to 7.9%. *Ulva* spp. also contained 4c7c10c13c16c19c-22:6 in the present study in low amounts (1.1–2.3%) in congruence with previous reports for *Ulva* spp. (0.1–1.44%) from Yellow Sea [50], Black Sea and Dardenelles [51], Iranian coast [43], southern Australian coast of Tasmania [4], Brazilian coast [46], and Chilean sub-Antarctic region [15]. However, Kumari and co-workers reported relatively higher amounts of 4c7c10c13c16c19c-22:6, approximately 0.7% to 3.4%, from twelve species of fresh *Ulva* thalli collected during March–October 2011 [14] and 2.15% to 6.05% from shade-dried *Ulva* species [11] collected during January–April 2008 from the Indian coast. Such large variations in 4c7c10c13c16c19c-22:6 content in *Ulva* spp. can be due to inter-specific variation, different sampling sites, season, and other environmental factors [14,28,52]. 

*C*. *papillatum* belonging to the Bryopsidales displayed a distinctly different fatty acid profile from those observed for *Ulva* spp. (Table 2). Specifically, the SFAs content was 1.3 to 1.6- fold higher, mainly due to higher levels in 16:0 (1.2 to 1.5-fold) and 9c-18:1 (*n*9) (1.2 to 3.1-fold), while the content of PUFAs was significantly lower, especially 9c12c-18:2 (2.2 to 5-fold) and 9c12c15c-18:3 (4.8 to 6.3-fold) as compared with *Ulva* spp. These differences in FA profiles between *Ulva* spp. and *C*. *papillatum* may be due to genotypic differences as well as different time of sampling. Similar FA profiles with high SFA, 16:0 and low 9c12c-18:2, 9c12c15c-18:3, and minor amounts of C20 PUFAs have been reported for *C*. *dwarkense* from Gujarat coast, India [14] and *C*. *bursa* from Adriatic Sea, Croatia [29]. However, other studies have detected appreciable amounts of 16:0 (20.9–38.7%), low MUFAs and high PUFAs with C16 PUFAs (16.8–18.9%), C18 PUFAs (25.4–29.5%), and C20 PUFAs (6.4–14.6%) in different *Codium* species including *C*. *fragile*, *C*. *geppi*, *C*. *papillatum*, *C*. *tomentosum,* and *Codium* sp. across different regions across the world [4,39,48,49,53]. Previously, Dembitsky et al. [54] demonstrated large variations in the FA content of the genus *Codium*, depending on the species, the season, and the geographic origin of the sample.

#### 3.1.2. Rhodophyta

We investigated only one red seaweed, *Chondria* sp., belonging to the order Ceramiales (Table 2). *Chondria* sp. had the highest SFA and the lowest MUFA and PUFA contents among all the seaweeds investigated in the present study. The highest content of SFA was mainly due to high contents of 16:0 (63.9%) and 14:0 (11.7%), in line with the previous report of Govenkar and Wahidullah [55]. These authors reported high SFAs (80.73%), of which 16:0 accounted for 74.3% and low MUFAs (19.27%), while no PUFA was detected in *Chondria armata* [55]. High 14:0 contents (6.8–13.4%) are characteristic of the seaweeds of the order Ceramiales [11,14]. In contrast to our findings, 5c8c11c14c-20:4 and 5c8c11c14c17c-20:5 have been reported in high amounts together contributing to 43.3% in *Chondria dasyphylla* and 23.8% in *Chondria decipiens* from the Sea of Japan, respectively [56]. Vaskovsky et al. [50] reported low 16:0 (27.7%), 9c-18:1 (6.4%), C18 PUFAs <1%, and high C20 PUFAs (45.7%) in *Chondria capillaris* from the Yellow Sea. Stefanov et al. [57] showed large variations in the FA contents of *C*. *capillaris* collected from the Black Sea and Lake Pomorie (Bulgaria), especially in SFAs (42.4–60.2%) and PUFA contents (11.7–27.2%).

#### 3.1.3. Ochrophyta

The FA compositions of four brown seaweeds belonging to the orders Dictyotales, Ectocarpales, and Fucales are given in Table 2. While the contents of individual FAs differed significantly between different individual species, a common trait of different brown seaweed FA profiles was high content of 14:0, 16:0, and 9c-18:1 together accounting for 72.6% to 76.3%. In general, for brown seaweeds investigated in the present study, we found higher SFA contents (61.3–71.3%), similar MUFAs (20.5–26.0%), but lower PUFAs (7.4–17.6%) compared with the same or related brown seaweed species reported from different parts of the world [4,11,14,23,24,37,39,45,46,47,48,58]. Interestingly, Rohani-Gadhikolaei et al. [35] reported comparable FA profiles with high SFAs (51.9–55.2%), MUFAs (27.5–32.9%), and low PUFAs (15.3–17.4%), especially low 5c8c11c14c-20:4 and 5c8c11c14c17c-20:5 (together accounting for only 2.9–5.8%), for brown seaweeds including *Sargassum* spp. and *Colpomenia sinuosa* from the Iranian coast. It is worth noting that the two Ectocarpales species investigated by us, *I*. *stellata* and *F*. *indica*, had distinct FA compositions, which is in line with the large variations in FA profiles reported for different species of Ectocarpales [4,14,39]. *I*. *stellata* had higher contents of SFAs (1.2-fold), 12:0 (9.6-fold), 16:0 (1.1-fold), 18:0 (1.5-fold), 22:0 (10.5-fold), but low 14:0 (2.4-fold), 9c-16:1 (3.0-fold) and PUFAs (2.4-fold) as compared with *F*. *indica* (Table 2). *S*. *aquifolium* (Fucales) had lower 14:0 (1.4-fold) and 6c9c12c15c-18:4 contents (3.8-fold) as compared with *P*. *boergesenii* (Dictyotales) as reported previously for Fucales members [4,11,14,24,45]. Furthermore, brown seaweeds differ from red and green seaweeds based on their predominantly higher amounts of both C18 and C20 PUFAs (both DW and FW basis) [4,11,14,24,47,48,58]. Kumari et al. [14] reported FA profiles of 24 brown seaweeds exhibiting higher PUFA contents (23.7–58.0%) and further differentiated them into three groups based on the relative contents of C18 and C20 PUFAs. The first group included brown seaweeds containing higher C18 PUFAs (1.1 to 1.2-fold) such as *Padina* spp., *Sirophysalis trinodis,* and *Feldmannia mitchelliae*. The second group included species containing higher C20 PUFAs such as *Sargasuum* spp., *Dictyota* spp. (*D*. *pinnatifida*, *D*. *bartayresiana*, *D*. *dichotoma*), and *Hormophysa cuneiformis*, while the third group consisted of *Dictyopteris delicatula*, *Canistrocarpus cervicornis,* and *Dictyota ciliolata* containing equal amounts of C18 and C20 PUFAs. Despite low PUFA contents determined in brown seaweeds in our study, *P. boergesenii*, *I*. *stellata* and *F*. *indica* had 1.8 to 2.9-fold higher C18 PUFAs than C20 PUFAs, while *S*. *aquifolium* had 1.2-fold higher C20 PUFA levels than C18 PUFAs in congruence with the previous reports about *Padina* spp., *Sargassum* spp. and related brown seaweeds [11,14]. Similar higher contents of C20 PUFAs than those of C18 PUFAs (1.02 to 3.8-fold; due to higher 5c8c11c14c-20:4 and 5c8c11c14c17c-20:5 contents) were reported for different *Sargassum* spp. collected from different regions of the world [24,39,48,59]. On the contrary, Verma et al. [45] reported higher C18 PUFAs in all the brown seaweeds studied including *Padina tetrastomatica*, *Spatoglossum asperum*, *Feldmannia marginatum*, *I*. *stellata,* and *Sargassum linearifolium* due to higher 9c12c-18:2contents (17.16–23.15% TFA) while 5c8c11c14c-20:4 and 5c8c11c14c17c-20:5 were detected in minor amounts.

Overall, a substantial variation was observed in the individual FA contents of the same and related species of the same genus among all green, red, and brown seaweeds in our study, which is also reflected in the literature from different regions of the world [4,24,39,45,47,48,52]. These variations are due to species-specific variations, different geographical locations, and environmental factors (temperature, light, salinity, nutrients) [25,28,30,46,59]. Thus, it becomes necessary to screen different seaweeds (both wild and cultivated) from different regions for their FA contents and to monitor them across different seasons to determine the suitable period of harvest for seaweed valorization. The effect of different seasons or other environmental factors on FA composition of seaweeds were not studied in the present study but will be an objective of our future research. Additionally, there can be variations in FA contents of same or related species in literature due to different extraction and derivatization methods employed by researchers [25,60], but it is beyond the scope of this study to compare such FA variations.

### 3.2. Fatty Acid Chemotaxonomy

**Figure 1:**
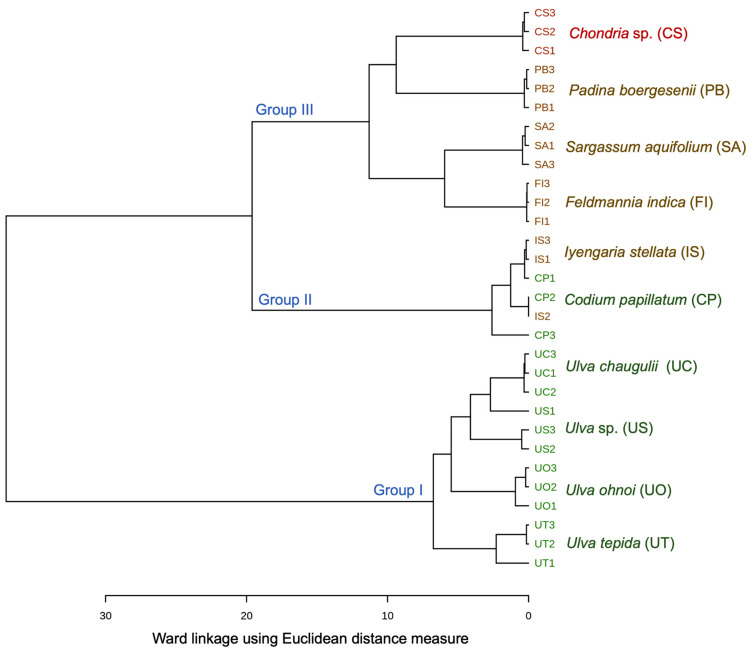
Dendrogram obtained from hierarchical clustering of seaweeds samples using Ward linkage with Euclidean distance.

Hierarchical clustering was performed on the FA data matrix (Supplementary datasheet S1) to evaluate the chemotaxonomic relationships between different species at different taxonomic levels. A few FAs, namely, 10:0, 13:0, 24:0, 9c-14:1, 10c-15:1, 10c-17:1, 9t-18:1, 11c-20:1, 13c-22:1, 15c-24:1, 9t12t-18:2, 13c16c-22:2, and 11c14c17c-20:3 were excluded from this FA data matrix due to their insignificant amounts and lack of correlation with the data matrix since such variables often lead to misclassification of species. The dendrogram obtained from Ward hierarchical clustering grouped the seaweed samples into three demarcated clusters (Figure 1).

Ward linkage is an agglomerative clustering algorithm which starts with n singleton clusters (each consisting of one element of the data set) and merges two clusters based on similarity measure. All of the *Ulva* species (Ulvales) were grouped together in group I while *C*. *papillatum* (Bryopsidales) was grouped with *I*. *stellata* (Ectocarpales) in group II. The single red alga investigated in this study, *Chondria* sp., belonging to Ceramiales, was grouped together with brown seaweeds *P*. *boergesenii* (Dictyotales), *S*. *aquifolium* (Fucales), and *F*. *indica* (Ectocarpales) in group III. Kumari et al. [14] also showed that Bryopsidales are grouped separately from Ulvales and the latter generally aligns with Ulotrichales, forming the Ulvales-Ulotrichales clade [14]. However, a greater number of replicates as well as species belonging to the genus *Codium* and *Iyengaria* are required to resolve their misclassification based on FAs, as observed in our study. Further, group I can be sub-divided into two sub-groups, consisting of *U*. *tepida* in one, and *U*. *ohnoi*, *Ulva* sp. and *U*. *chaugulii* in another sub-group. Similarly, group III can be further sub-divided into three sub-groups, the first comprising of *F*. *indica* and *S*. *aquifolium*, the second of *P*. *boergesenii,* which was closely related to *Chondria* sp., forming the third sub-group. Similarly, the species belonging to the genera *Padina* and *Sargassum* were grouped in different sub-groups based on their FA profiles [11,14,45] as well as different clades based on their molecular data [61]. However, for adequate comparison of inter-relationships between different groups deduced from FA composition with the clades inferred from genomic data, extensive sampling effort with samples belonging to the same genus as well as same class or orders are imperative.

Thus, our study displayed that FA traits are conserved in seaweeds at higher ordinal levels of families, orders, and phyla, in line with the previous findings [4,11,14,16,17,45]. FA signatures could be potential tools for understanding the chemotaxonomic relationships among different seaweed species, but require proper sampling. Otherwise, higher variations in FA contents at the levels of genus or species may pose difficulty in discriminating species in the absence of adequate taxon sampling and replicates, as observed in our study for *C*. *papillatum* and *I*. *stellata*.

### 3.3. Nutritional Assessment for Seaweed Valorization

Our study revealed that *Ulva* species are rich sources of nutritionally important PUFAs with their unsaturation indices (UI) varying from 119.21 ± 0.45 (*U*. *ohnoi*) to 133.28 ± 1.65 (*U*. *tepida*) (Table 2) in congruence with the UI values reported in the literature for different species of the genus *Ulva* [11,14,62]. The UI values for all other species in our study were low, varying from 28.63 ± 0.84 (*Chondria* sp.) to 78.91 ± 0.35 (*F*. *indica*) in agreement with lower PUFA contents in these species.

Further, we conducted a principal component analysis (PCA) (without rotation) and hierarchical clustering based on and the nutritional indices data matrix (Supplementary datasheet S2) to identify potential seaweeds that can be valorized for nutritional and functional food applications. We obtained a KMO value of 0.726 and a significant level for the Bartlett’s test (Supplementary Table S1) for the nutritional indices data matrix, suggesting that nutritional indices variables were highly correlated.

**Figure 2:**
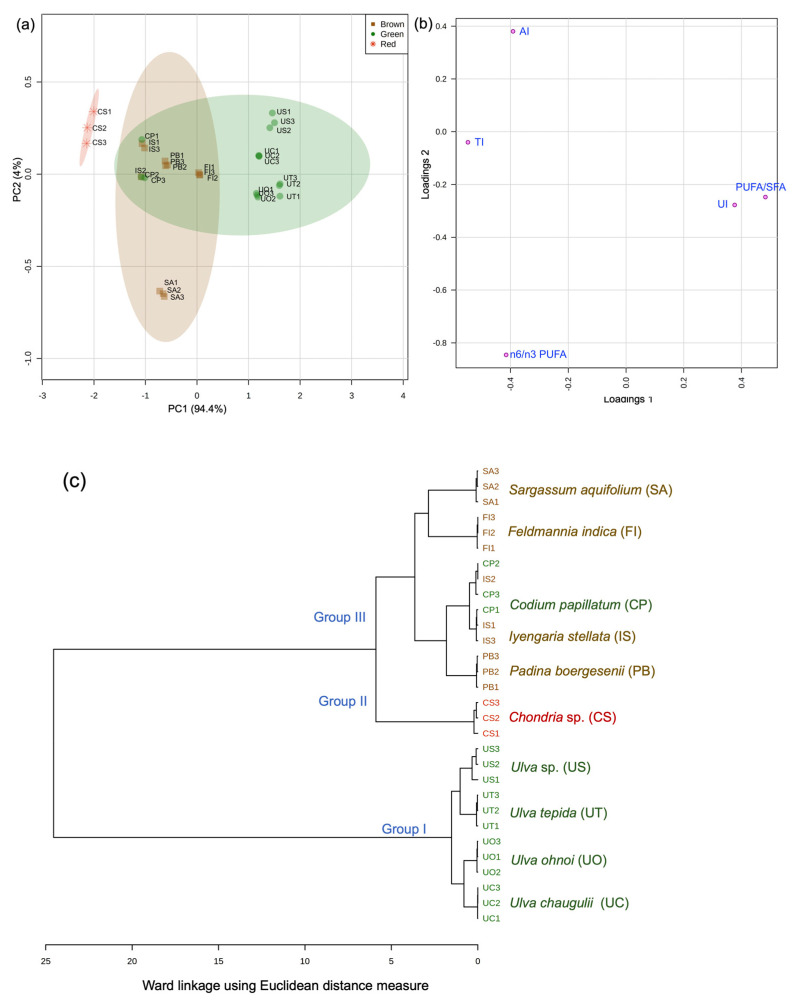
Principal component analysis (PCA) and hierarchical clustering of seaweeds based on nutritional indices. (**a**) PCA scores plot and (**b**) PCA loadings plot and (**c**) dendrogram obtained from hierarchical clustering using Ward linkage and Euclidean distance measures. Seaweed species are labeled as mentioned in Table 1. Abbreviations: AIatherogenic index, PUFApolyunsaturated fatty acids, SFAsaturated fatty acid, STAstearidonic acid, TIThrombogenic index, UIunsaturation index.

The principal components were extracted based on scree plot (Supplementary Figure S1) and the first two principal components, which also presented the maximum explained variance, were used for generating scores and loading plot. PCA of nutritional indices data matrix explained 98.4% of variations (PC1-94.4% and PC2-4%) (Figure 2a). The discriminant variables along PC1 were PUFA/SFA, UI, and TI, and along PC2 were AI and *n*6/*n*3 PUFA (Figure 2b). The loadings plot displayed that PUFA/SFA and UI were highly positively correlated, while both these were negatively correlated with TI. Similarly, AI was negatively correlated with *n*6/*n*3 PUFAs. Further, PC2 (*Y*-axis) separated all the *Ulva* species from brown and red seaweeds owing to their higher loadings of PUFA/SFA and UI, while *C*. *papillatum* was positioned along with the brown seaweeds due to its lower contents of UI and PUFA/SFA. *S*. *aquifolium* was found to be the outlier, separated from rest of the brown seaweeds by *X*-axis due to its high loadings of *n*6/*n*3 PUFAs. *Chondria* sp. was separated from the rest of green and brown seaweeds due to its higher loadings of TI and AI in line with its exceptionally high TI and AI contents (Table 2).

The dendrogram obtained from hierarchical clustering of nutritional indices data revealed three demarcated clusters (Figure 2c). All of *Ulva* spp. (containing high UI and PUFA/SFA) were clustered together in Group I, like the Ulvales clade deduced from the FA data matrix (Figure 1). Contrary to our previous results, where *Chondria* sp. was grouped with other brown seaweeds in group III (Figure 1), here, *Chondria* sp. formed a separate clade, group II. *C*. *papillatum* was grouped with brown seaweeds in group III, sharing the sub-clade with *I*. *stellata* and *P*. *boergesonii*.

PUFAs are essential biomolecules to human health since their consumption is associated with decreased risk of cardiovascular and inflammatory diseases as well as cancer [18,19,20,63,64]. 9c12c15c-18:3 is a precursor of 5c8c11c14c17c-20:5 as well as 4c7c10c13c16c19c-22:6 and has anticancer, antiosteoporotic, antioxidant, anti-inflammatory, as well as coronary and neuronal protective effects [65]. 4c7c10c13c16c19c-22:6 is essential for visual and neurological development in infants while 5c8c11c14c-20:4 and 5c8c11c14c17c-20:5 are precursors of prostaglandins, thromboxanes, and other eicosanoids that influence inflammation processes and immune reactions [18,63]. Free PUFAs also have biological effects including induction of an oxidative burst, oxylipin biosynthesis, and induction of resistance against pathogens in seaweeds such as the brown algal kelps *Laminaria digitata* and *Macrocystis pyrifera* [66]. The PUFA/SFA ratio, which is an important parameter to assess the nutritional quality of the lipid fraction of food, should be ≥0.4 [67]. In this study, the PUFA/SFA values were in accordance with the nutritional guidelines only for *Ulva* species (0.57–0.80). However, much higher PUFA/SFA values (≥0.4) have been reported for species of the genera *Ulva*, *Codium*, *Sargassum,* and *Padina* in previous reports [4,14,15,24,25,34,43,46,49]. The low PUFA/SFA ratio in our study may be due the warm environment of the Arabian Gulf, in agreement with the reports that seaweeds of temperate regions tend to feature a higher degree of unsaturation in their fatty acid composition [46,59,62,68]. High SFA content in tropical seaweeds may be related to their physiological adaptation to warm temperatures, while high PUFA content in cold water may facilitate thermo-adaptive regulation of membrane lipid fluidity [59,69]. All the species investigated in the present study had health-promoting *n*6/*n*3 ratios ranging from 0.33 ± 0.02:1 (*Ulva* sp.) to 2.94 ± 0.03:1 (*S*. *aquifolium*) (Table 2) in line with the World Health Organization (WHO) recommendations of an *n*6/*n*3 ratio of 5:1 [19,20,70]. The atherogenic indices (AI) varied from 0.8 ± 0.01 (*U*. *tepida*) to 2.52 ± 0.04 (*P*. *boergesenii*), while thrombogenic indices (TI) varied from 0.49 ± 0.01 (*U*. *tepida*) to 5.17 ± 0.15 (*I*. *stellata*), except for *Chondria* sp., which had higher AI and TI values (Table 2). Low AI and TI < 3 have been reported for different green, red, and brown seaweeds in the literature [2,14,24,25,49]. Recently, Chen and Liu [23] compared the nutritional indies of numerous seaweeds reported in the literature with those of plant oils, fish, and dairy products. Accordingly, AI and TI values obtained in our study for all seaweeds except *Chondria* sp. (Table 2) were comparable to those of fish (AI—0.37–1.22, TI—0.14–0.87), shrimps (AI—0.71–0.82, TI—0.21–0.30), and dairy products (AI—1.42–5.13, TI—0.39–5.04) [23]. There is no recommended level of AI and TI in food products, but the consumption of foods with low AI and TI indices is helpful in reducing the risk of coronary heart diseases [23].

Overall, most of the seaweeds investigated in our study had health-beneficial *n*6/*n*3, AI, and TI values, but only *Ulva* spp. had higher UI and the recommended PUFA/SFA ratio. The multivariate analysis of nutritional indices clearly supported our findings and helped in assessing the nutritional potential of seaweeds from Arabian Gulf. Nevertheless, *Ulva* spp. are not only rich in essential PUFAs, but also contain high amounts of macro- and micronutrients as reported previously [71]. In addition, the nutritional value of *Ulva* species in terms of carbohydrates, protein, and fatty acids (especially PUFA content) has been reported to be comparable to some vegetables, nuts, and grains [2,23,72] and it has been consumed traditionally for centuries in many Asian countries [1,2,4,25,72].

## 4. Final Conclusions

Our study revealed that seaweeds from the Arabian Gulf exhibit typical FA profiles of warm waters with relatively high SFA and low PUFA contents. The green, red, and brown seaweeds exhibit species-specific significant differences in FA contents, but trends of FA profiles were conserved at different taxonomic ranks of genus, class, and order within each phyla. Among all the species investigated, *Ulva* spp. are the most suitable candidates for developing low-fat foods with PUFA-rich nutraceuticals or utilization in functional food for human consumption and animal feed due to their health beneficial PUFA/SFA, *n*6/*n*3, AI, and TI values. However, proper valorization of *Ulva* species for commercial utilization will require a temporal, spatial, and seasonal consistency in FA contents. Future studies for understanding the environmental and seasonal impacts on FA profiles of *Ulva* spp. from the Kuwait region will facilitate selecting the correct harvest time for obtaining high PUFA yields.

## Figures and Tables

**Table 1:** Locations of macroalgal sampling sites along the Kuwait coastline.

S. No.	Species	Abbreviations (*)	Phylogenetic Affinity	Herbarium Code	Date	Location	Coordinates	Offshore Seawater Surface Temperature (°C)
Chlorophyta								
1	*Ulva* sp.	US	Ulvaceae, Ulvales, Ulvophyceae	Doh010221-1	01/02/2021	Ras Ushairij	29°23′00.7″ N 47°49′50.9″ E	14.8–15.0
2	*Ulva chaugulii* M.G.Kavale and M.A.Kazi	UC	Ulvaceae, Ulvales, Ulvophyceae	Doh010221-2	01/02/2021	Ras Ushairij	29°23′00.7″ N 47°49′50.9″ E	14.8–15.0
3	*Ulva tepida* Masakiyo and S.Shimada	UT	Ulvaceae, Ulvales, Ulvophyceae	Doh010221-5	01/02/2021	Ras Ushairij	29°23′00.7″ N 47°49′50.9″ E	14.8–15.0
4	*Ulva ohnoi* M.Hiraoka and S.Shimada	UO	Ulvaceae, Ulvales, Ulvophyceae	Doh010221-3	01/02/2021	Ras Ushairij	29°23′00.7″ N 47°49′50.9″ E	14.8–15.0
5	*Codium papillatum* C.K.Tseng and W.J. Gilbert	CP	Codiaceae, Bryopsidales, Ulvophyceae	ABUH030618-2	3/06/2018	Abu Al Hasaniya	29°12′19.4″ N 48°06′41.5″ E	30.2–30.3
Rhodophyta								
6	*Chondria* sp. C. Agardh	CS	Rhodomelaceae Ceramiales, Florideophyceae	BNA260518-1	26/05/2018	Bnaider Beach	28°47′01.5″ N 48°17′50.6″ E	27.8–27.9
Ochrophyta								
7	*Iyengaria stellata* (Børgesen) Børgesen	IS	Scytosiphonaceae, Ectocarpales, Phaeophyceae	ABUH060618-1	27/05/2018	Abu Al Hasaniya	29°12′19.4″ N 48°06′41.5″ E	30.2–30.3
8	*Feldmannia indica* (Sonder) Womersley and A. Bailey	FI	Acinetosporaceae Ectocarpales, Phaeophyceae	ABUH030618-1	3/06/2018	Abu Al Hasaniya	29°12′19.4″ N 48°06′41.5″ E	30.2–30.3
9	*Padina boergesenii* Allender and Kraft	PT	Dictyotaceae, Dictyotales, Phaeophyceae	ABUH270518-1	27/05/2018	Abu Al Hasaniya	29°12′19.4″ N 48°06′41.5″ E	30.2–30.3
10	*Sargassum aquifolium* (Turner) C. Agardh	SA	Sargassaceae, Fucales, Phaeophyceae	ABUH270518-2	27/05/2018	Abu Al Hasaniya	29°12′19.4″ N 48°06′41.5″ E	30.2–30.3

**Table 2:** Fatty acid composition (% of total fatty acid methyl esters) of different macroalgal species, expressed as means ± SD (*n* = 3).

Fatty Acids	*Ulva* sp.	*Ulva chaugulii*	*Ulva ohnoi*	*Ulva tepida*	*Codium* *papillatum*	*Chondria* sp.	*Iyengaria* *stellata*	*Feldmannia* *indica*	*Padina* *boergesenii*	*Sargassum* *aquifolium*
10:0	nd	nd	nd	nd	0.3 ± 0.01 ^c^	0.5 ± 0.1 ^a^	0.3 ± 0.01 ^c^	0.4 ± 0.003 ^b^	0.3 ± 0.03 ^c^	0.3 ± 0.02 ^c^
12:0	0.2 ± 0.1 ^f^	0.4 ± 0.02 ^de^	1.6 ± 0.2 ^b^	1.2 ± 0.1 ^c^	1.9 ± 0.01 ^a^	0.5 ± 0.01 ^d^	1.9 ± 0.04 ^a^	0.2 ± 0.02 ^f^	1.3 ± 0.1 ^c^	0.4 ± 0.1 ^de^
13:0	0.1 ± 0.05 ^b^	nd	nd	nd	0.01 ± 0.001 ^c^	0.2 ± 0.02 ^a^	0.01 ± 0.001 ^c^	0.01 ± 0.001 ^c^	0.04 ± 0.04 ^c^	nd
14:0	1.3 ± 0.11 ^g^	1.7 ± 0.02 ^fg^	2.5 ± 0.04 ^e^	2.1 ± 0.05 ^ef^	3.3 ± 0.07 ^d^	11.7 ± 0.42 ^a^	3.2 ± 0.06 ^d^	7.6 ± 0.04 ^b^	7.5 ± 0.05 ^b^	5.6 ± 0.13 ^c^
15:0	0.2 ± 0.04 ^e^	0.2 ± 0.01 ^e^	0.3 ± 0.001 ^d^	0.7 ± 0.02 ^ab^	0.1 ± 0.01 ^f^	0.8 ± 0.04 ^a^	0.1 ± 0.02 ^f^	0.5 ± 0.001 ^c^	0.7 ± 0.01 ^ab^	0.4 ± 0.03 ^d^
16:0	43.5 ± 5.0 ^de^	44.6 ± 0.2 ^cde^	41.3 ± 0.4 ^e^	35.7 ± 0.6 ^f^	53.9 ± 0.6 ^b^	63.9 ± 0.5 ^a^	53.7 ± 0.8 ^b^	49.0 ± 0.2 ^bcd^	49.2 ± 0.3 ^bc^	49.1 ± 0.5 ^bc^
17:0	0.1 ± 0.003 ^c^	0.2 ± 0.05 ^bc^	0.2 ± 0.1 ^bc^	0.1 ± 0.003 ^c^	0.2 ± 0.02 ^bc^	0.4 ± 0.02 ^b^	0.2 ± 0.01 ^bc^	0.3 ± 0.002 ^bc^	5.9 ± 0.2 ^a^	0.1 ± 0.05 ^bc^
18:0	3.4 ± 2.0 ^ab^	1.1 ± 0.05 ^c^	1.7 ± 0.17 ^bc^	1.3 ± 0.13 ^bc^	3.2 ± 0.19 ^abc^	4.5 ± 0.37 ^a^	3.0 ± 0.2 ^abc^	1.9 ± 0.03 ^bc^	2.3 ± 0.1 ^abc^	3.2 ± 0.01 ^abc^
20:0	1.2 ± 0.3 ^ab^	1.2 ± 0.01 ^abc^	0.9 ± 0.01 ^abcd^	1.4 ± 0.03 ^a^	0.7 ± 0.5 ^bcd^	0.5 ± 0.01 ^d^	0.9 ± 0.1 ^abcd^	0.5 ± 0.001 ^d^	0.6 ± 0.02 ^cd^	0.9 ± 0.02 ^abcd^
22:0	1.6 ± 0.01 ^c^	1.7 ± 0.01 ^c^	4.5 ± 0.1 ^b^	2.0 ± 0.04 ^c^	5.6 ± 0.4 ^a^	0.2 ± 0.04 ^de^	5.6 ± 0.31 ^a^	0.5 ± 0.01 ^de^	0.1 ± 0.0 ^e^	0.8 ± 0.02 ^d^
24:0	nd	0.5 ± 0.004 ^c^	1.1 ± 0.02 ^b^	0.02 ± 0.001 ^de^	2.7 ± 0.4 ^a^	0.3 ± 0.03 ^c^	2.4 ± 0.3 ^a^	0.3 ± 0.02 ^c^	0.3 ± 0.03 ^c^	0.4 ± 0.1 ^c^
ΣSFAs	51.6 ± 3.0 ^e^	51.4 ± 0.11 ^e^	54.1 ± 0.6 ^e^	44.5 ± 0.8 ^f^	72.0 ± 0.9 ^b^	83.3 ± 0.3 ^a^	71.3 ± 0.5 ^b^	61.3 ± 0.1 ^d^	68.0 ± 0.4 ^c^	61.2 ± 0.5 ^d^
9c-14:1	0.2 ± 0.04 ^a^	nd	nd	nd	0.02 ± 0.003 ^c^	nd	0.02 ± 0.003 ^c^	0.03 ± 0.004 ^bc^	nd	0.1 ± 0.01 ^b^
10c-15:1	nd	nd	0.04 ± 0.003 ^a^	0.02 ± 0.001 ^b^	nd	nd	nd	nd	nd	nd
9c-16:1	7.3 ± 2.2 ^a^	6.4 ± 0.03 ^ab^	3.9 ± 0.04 ^cd^	1.4 ± 0.02 ^d^	1.6 ± 0.04 ^d^	5.4 ± 0.1 ^abc^	1.5 ± 0.1 ^d^	4.4 ± 0.1 ^bc^	3.2 ± 0.3 ^cd^	4.6 ± 0.1 ^bc^
10c-17:1	0.3 ± 0.2 ^bc^	0.7 ± 0.001 ^a^	0.1 ± 0.003 ^c^	nd	0.04 ± 0.002 ^c^	nd	0.04 ± 0.002 ^c^	0.5 ± 0.003 ^ab^	0.1 ± 0.02 ^c^	0.1 ± 0.02 ^c^
9t-18:1	0.1 ± 0.06 ^b^	3.0 ± 0.03 ^a^	0.7 ± 0.1 ^b^	3.2 ± 0.1 ^a^	0.1 ± 0.05 ^b^	nd	0.1 ± 0.04 ^b^	0.04 ± 0.0004 ^b^	0.1 ± 0.002 ^b^	0.1 ± 0.01 ^b^
9c-18:1	5.9 ± 3.0 ^de^	8.9 ± 0.03 ^d^	6.1 ± 0.01 ^de^	15.7 ± 0.1 ^c^	18.1 ± 0.7 ^ab^	4.8 ± 0.1 ^e^	19.4 ± 0.5 ^ab^	16.0 ± 0.04 ^bc^	16.8 ± 0.8 ^abc^	19.8 ± 0.2 ^a^
11c-20:1	1.5 ± 1.2 ^a^	0.1 ± 0.1 ^b^	0.2 ± 0.002 ^ab^	0.2 ± 0.1 ^ab^	0.1 ± 0.03 ^b^	0.4 ± 0.01 ^ab^	0.1 ± 0.03 ^b^	0.03 ± 0.0003 ^b^	0.1 ± 0.01 ^b^	0.9 ± 0.03 ^ab^
13c-22:1	0.2 ± 0.01 ^cd^	0.2 ± 0.05 ^cd^	0.6 ± 0.1 ^a^	0.2 ± 0.06 ^cd^	0.02 ± 0.001 ^e^	0.20 ± 0.01 ^cd^	0.02 ± 0.001 ^e^	0.1 ± 0.01 ^de^	0.2 ± 0.1 ^cd^	0.4 ± 0.02 ^b^
15c-24:1	0.5 ± 0.1	nd	nd	nd	nd	nd	nd	nd	nd	nd
ΣMUFAs	15.8 ± 7.0 ^bc^	19.4 ± 0.1 ^ab^	11.5 ± 0.9 ^b^	20.7 ± 0.2 ^ab^	20.0 ± 0.7 ^ab^	10.8 ± 0.2 ^c^	21.2 ± 0.5 ^ab^	21.1 ± 0.04 ^ab^	20.5 ± 0.8 ^ab^	26.0 ± 0.3 ^a^
9t12t-18:2	nd	nd	nd	nd	0.3 ± 0.1 ^ab^	0.5 ± 0.1 ^a^	0.3 ± 0.11 ^b^	0.003 ± 0.001 ^c^	0.3 ± 0.1 ^ab^	nd
9c12c-18:2	5.2 ± 0.5 ^d^	7.0 ± 0.1 ^c^	11.6 ± 0.1 ^a^	8.6 ± 0.1 ^b^	2.3 ± 0.3 ^f^	1.6 ± 0.1 ^f^	2.0 ± 0.3 ^f^	3.7 ± 0.01 ^e^	3.3 ± 0.1 ^e^	3.3 ± 0.1 ^e^
6c9c12c-18:3	1.5 ± 0.22 ^b^	1.5 ± 0.03 ^b^	0.9 ± 0.06 ^c^	1.8 ± 0.05 ^a^	0.4 ± 0.03 ^d^	0.2 ± 0.01 ^d^	0.4 ± 0.03 ^d^	0.4 ± 0.01 ^d^	0.4 ± 0.02 ^d^	0.2 ± 0.01 ^d^
9c12c15c-18:3	13.5 ± 1.6 ^a^	11.0 ± 0.04 ^b^	11.4 ± 0.1 ^b^	13.4 ± 0.2 ^a^	2.3 ± 0.2 ^d^	0.5 ± 0.1 ^d^	2.3 ± 0.1 ^d^	5.1 ± 0.02 ^c^	2.2 ± 0.1 ^d^	1.7 ± 0.04 ^d^
6c9c12c15c-18:4	7.4 ± 1.1 ^ab^	6.4 ± 0.02 ^bc^	5.6 ± 0.04 ^c^	7.9 ± 0.1 ^a^	0.1 ± 0.02 ^e^	0.3 ± 0.03 ^e^	0.1 ± 0.02 ^e^	2.0 ± 0.004 ^d^	2.2 ± 0.1 ^d^	0.6 ± 0.01 ^e^
8c11c14c-20:3	0.2 ± 0.1 ^de^	0.3 ± 0.1 ^bcd^	0.4 ± 0.01 ^bc^	0.2 ± 0.06 ^de^	0.1 ± 0.01 ^e^	0.2 ± 0.01 ^de^	0.1 ± 0.01 ^e^	0.4 ± 0.01 ^bc^	0.5 ± 0.02 ^b^	1.4 ± 0.05 ^a^
11c14c17c-20:3	0.1 ± 0.04 ^b^	nd	nd	0.1 ± 0.02 ^b^	0.3 ± 0.03 ^a^	nd	0.3 ± 0.02 ^a^	0.1 ± 0.004 ^b^	0.1 ± 0.01 ^b^	0.1 ± 0.003 ^b^
5c8c11c14c-20:4	0.7 ± 0.03 ^cde^	0.7 ± 0.01 ^cde^	0.7 ± 0.02 ^cde^	0.4 ± 0.1 ^e^	1.1 ± 0.1 ^cd^	1.3 ± 0.06 ^cd^	1.1 ± 0.08 ^cd^	4.0 ± 0.02 ^a^	2.0 ± 0.1 ^b^	4.3 ± 0.1 ^a^
13c16c-22:2	0.6 ± 0.1 ^ab^	nd	nd	0.4 ± 0.01 ^bc^	0.7 ± 0.1 ^a^	0.2 ± 0.01 ^de^	0.6 ± 0.1 ^a^	0.3 ± 0.01 ^cd^	0.1 ± 0.01 ^e^	0.3 ± 0.02 ^cd^
5c8c11c14c17c-20:5	1.4 ± 0.0 ^ab^	1.2 ± 0.01 ^bc^	1.5 ± 0.04 ^a^	0.8 ± 0.1 ^d^	0.4 ± 0.1 ^e^	0.8 ± 0.1 ^d^	0.3 ± 0.1 ^e^	1.5 ± 0.02 ^a^	0.3 ± 0.02 ^e^	0.9 ± 0.1 ^cd^
4c7c10c13c16c19c-22:6	1.9 ± 0.2 ^b^	1.1 ± 0.1 ^c^	2.3 ± 0.1 ^a^	1.8 ± 0.1 ^b^	nd	nd	0.2 ± 0.11 ^d^	0.1 ± 0.003 ^d^	nd	nd
ΣPUFAs	32.4 ± 34.0 ^ab^	29.2 ± 0.1 ^b^	34.4 ± 0.2 ^a^	35.4 ± 0.5 ^a^	7.9 ± 0.5 ^ef^	5.6 ± 0.1 ^f^	7.4 ± 0.4 ^f^	17.5 ± 0.1 ^c^	11.4 ± 0.5 ^de^	12.9 ± 0.3 ^d^
ΣC18 PUFAs	27.6 ± 3.3 ^a^	25.8 ± 0.1 ^a^	29.4 ± 0.2 ^a^	31.7 ± 0.4 ^a^	5.4 ± 0.3 ^cd^	3.2 ± 0.2 ^e^	5.0 ± 0.3 ^cd^	11.2 ± 0.04 ^b^	8.4 ± 0.3 ^bc^	5.8 ± 0.1 ^cd^
ΣC20 PUFAs	2.3 ± 0.3 ^bc^	2.2 ± 0.1 ^bc^	2.6 ± 0.03 ^b^	1.5 ± 0.5 ^e^	1.8 ± 0.1 ^cd^	2.3 ± 0.2 ^bc^	1.7 ± 0.04 ^cd^	6.0 ± 0.1 ^a^	2.9 ± 0.2 ^b^	6.7 ± 0.2 ^a^
*n*6/*n*3 PUFA	0.3 ± 0.02 ^e^	0.5 ± 0.002 ^e^	0.7 ± 0.002 ^de^	0.5 ± 0.002 ^e^	1.7 ± 0.2 ^b^	2.5 ± 0.3 ^a^	1.6 ± 0.2 ^b^	1.0 ± 0.003 ^cd^	1.4 ± 0.03 ^bc^	2.9 ± 0.03 ^a^
PUFA/SFA	0.6 ± 0.04 ^b^	0.6 ± 0.003 ^b^	0.6 ± 0.003 ^b^	0.8 ± 0.02 ^a^	0.1 ± 0.01 ^e^	0.1 ± 0.002 ^e^	0.1 ± 0.01 ^e^	0.3 ± 0.002 ^c^	0.2 ± 0.01 ^d^	0.2 ± 0.01 ^d^
UI	123.9 ± 6.3 ^b^	112.8 ± 0.5 ^c^	119.2 ± 0.5 ^bc^	133.3 ± 1.5 ^a^	42.3 ± 1.3 ^g^	28.6 ± 0.8 ^h^	42.2 ± 0.8 ^g^	78.9 ± 0.4 ^d^	55.8 ± 0.8 ^f^	67.7 ± 1.3 ^e^
AI	1.0 ± 0.2 ^de^	1.1 ± 0.01 ^de^	1.2 ± 0.03 ^d^	0.8 ± 0.01 ^e^	2.5 ± 0.1 ^b^	6.7 ± 0.7 ^a^	2.4 ± 0.06 ^b^	2.1 ± 0.01 ^bc^	2.5 ± 0.04 ^b^	1.6 ± 0.04 ^c^
TI	0.6 ± 0.04 ^e^	0.7 ± 0.003 ^e^	0.6 ± 0.002 ^e^	0.5 ± 0.01 ^e^	5.0 ± 0.2 ^b^	11.1 ± 0.8 ^a^	5.2 ± 0.2 ^b^	2.0 ± 0.01 ^d^	3.2 ± 0.1 ^c^	4.0 ± 0.2 ^c^

nd—not detected; ^a–g^ Values in a row for each fatty acids without a common superscript are significantly different between different seaweeds at *p* < 0.01.

## Data Availability

Data are contained within the article.

## Supplementary Materials

The following are available online at https://www.mdpi.com/article/10.3390/foods10102442/s1, Supplementary Figure S1: Scree plot generated from principal component analysis of nutritional indices data matrix. Supplementary Table S1: Kaiser-Meyer-Olkin and Bartlett’s test values obtained from factor analysis of nutritional indices data matrix. Supplementary datasheet S1: Fatty acid data matrix used for hierarchical clustering. Supplementary datasheet S2: Nutritional indices data matrix used for principal component and hierarchical clustering analysis for nutritional assessment.
