# Supplementary material for: Assessment of Arabian Gulf Seaweeds from Kuwait as Sources of Nutritionally Important Polyunsaturated Fatty Acids (PUFAs)
Source: Foods. 2021 Oct 14;10(10):2442. doi: 10.3390/foods10102442 (PMC8536129; doi:10.3390/foods10102442)
Supplement: Supplementary file 1 [file foods-10-02442-s001.zip › Supplementary Materials.pdf]

Article

Assessment of Arabian Gulf Seaweeds from Kuwait as Sources of Nutritionally Important Polyunsaturated Fatty Acids (PUFAs)

Hanan Al-Adilah [1,2], Tahani Khalaf Al-Sharrah [3], Dhia Al-Bader [4], Rainer Ebel [2], Frithjof Christian Küpper [1,2] and Puja Kumari [1,2,*]

[1] School of Biological Sciences, University of Aberdeen, Cruickshank Building, St. Machar Drive, Aberdeen AB24 3UU, UK; h.aladilah.17@abdn.ac.uk (H.A.-A.); fkuepper@abdn.ac.uk (F.C.K.)
[2] Marine Biodiscovery Centre, Department of Chemistry, University of Aberdeen, Aberdeen AB24 3UE, UK; r.ebel@abdn.ac.uk
[3] Environmental Pollution & Climate Program, Environment and Life Sciences Research Centre, Kuwait Institute for Scientific Research, P.O. Box 24885, Safat 13109, Kuwait; t.sharrah@kisr.edu.kw
[4] Plant Biology Program, Department of Biological Sciences, Faculty of Science, Kuwait University, P.O. Box 5969, Safat 13060, Kuwait; dhiaaibader@yahoo.com
* Correspondence: puja.kumari@abdn.ac.uk

Citation: Al-Adilah, H.; Al-Sharrah, T.K.; Al-Bader, D.; Ebel, R.; Küpper, F.C.; Kumari, P. Assessment of Arabian Gulf Seaweeds from Kuwait as Sources of Nutritionally Important Polyunsaturated Fatty Acids (PUFAs). *Foods* **2021**, *10*, 2442. https://doi.org/10.3390/foods10102442

Academic Editors: Clélia Neves Afonso, Leonel Pereira and Teresa Mouga

Received: 18 September 2021
Accepted: 12 October 2021
Published: 14 October 2021

Publisher's Note: MDPI stays neutral with regard to jurisdictional claims in published maps and institutional affiliations.

Copyright: © 2021 by the authors. Licensee MDPI, Basel, Switzerland. This article is an open access article distributed under the terms and conditions of the Creative Commons Attribution (CC BY) license (https://creativecommons.org/licenses/by/4.0/).

Supplementary Materials

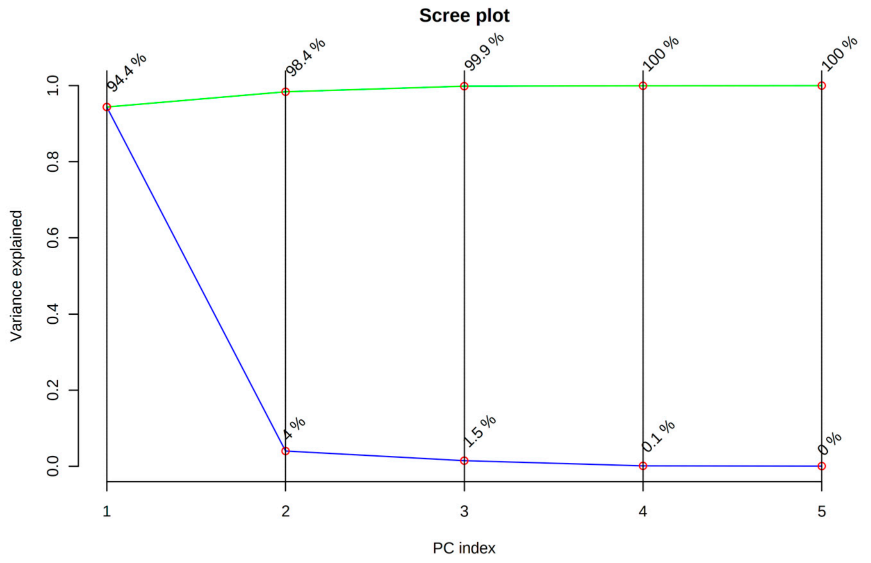

Supplementary Figure S1. Scree plot generated from principal component analysis of nutritional indices data matrix (using MetaboAnalyst 5.0).

Supplementary Table S1. Kaiser-Meyer-Olkin and Bartlet's test values obtained from factor analysis of nutritional indices data matrix using SPSS v22.

Kaiser-Meyer-Olkin Measure of Sampling Adequacy		0.726
Bartlett's Test of Sphericity	Approx. Chi-Square	368.126
	df	10
	Sig.	0.000

KMO value over 0.5 and a significance level for the Bartlett's test below 0.05 suggested substantial correlation in the data.
